# Work–Life Balance among Physicians in Jordan

**DOI:** 10.3390/medicina59050868

**Published:** 2023-04-29

**Authors:** Muayad Azzam, Manolia Al-Kubaisy, Mohammad A. Alshrouf, Joud Al Karmi, Hasan Alnawaiseh, Leith M. Mehyar, Sarah O. Ibrahim, Mohammad Abufaraj

**Affiliations:** 1 Faculty of Medicine, University of Jordan, Amman 11942, Jordan; azzam.muayad@outlook.com (M.A.);; 2 Division of Urology, Department of Special Surgery, Jordan University Hospital, University of Jordan, Amman 11942, Jordan; 3 Faculty of Medicine, Jordan University of Science and Technology, Irbid 22110, Jordan; 4 Department of Obstetrics and Gynecology, Jordan University Hospital, University of Jordan, Amman 11942, Jordan; 5 Department of Urology, The Medical University of Vienna, 1090 Vienna, Austria

**Keywords:** work–life balance, job satisfaction, life satisfaction, work–life conflict

## Abstract

Background and Objectives: We aimed to assess the work–life balance, job satisfaction, and life satisfaction and their correlates among physicians in Jordan. Materials and Methods: This study utilized an online questionnaire to collect information about work–life balance and related factors from practicing physicians in Jordan from August 2021 until April 2022. The survey consisted of 37 detailed self-report questions covering seven main categories: demographics, professional and academic information, impact of work on personal life, impact of personal life on work, work/personal life enhancement, the Andrew and Withney Job Satisfaction scale, and the Satisfaction with Life Scale developed by Diener et al. Results: The study included 625 participants. Overall, 62.9% were found to have a work–life conflict. The work–life balance score was negatively correlated with age, number of children, and years practicing medicine, while it was positively correlated with number of hours per week and number of calls. Regarding job and life satisfaction, 22.1% had a score indicating job dissatisfaction, while 20.5% disagreed with the statements indicating life satisfaction. Conclusion: Our study demonstrates that work–life conflict is highly prevalent among Jordanian physicians and highlights the significance of work–life balance in supporting physicians’ well-being and performance.

## 1. Introduction

Over the past 20 years, work–life balance (WLB), burnout, and physicians’ well-being have gained significant attention in the medical community [1]. Despite the wide adoption of the WLB concept, an agreed and precise definition has yet to be established [2]. It first emerged with the entry of women into the workforce and later expanded to include both genders [1]. As the work demands increased, individuals had to take on more responsibilities, which led to heavier workloads and longer working hours. This, combined with the rise of telecommunication and the internet, made managing relationships, personal lives, and family responsibilities more difficult [1]. Work–life conflict is now one of the most emerging psychosocial risk factors in the workplace, according to the European Agency for Safety and Health at Work Research [3].

Healthcare practitioners are more likely to suffer from work–life conflict due to their longer working hours than the general population [4]. In fact, about 50–60% of physicians experience symptoms of burnout in the United States, with a worsening trend in burnout and physicians’ satisfaction with the WLB over time [5,6]. Several studies have shown an increased risk of work–life conflict in physicians of younger age and in certain specialties [7,8]. These stressors directly affect their mental health and could lead to a myriad of issues, including depression, emotional exhaustion, anxiety, drug abuse, and even suicide [9]. In addition, studies have shown a connection between work–life conflict and medical errors [10]. Despite numerous studies on discussing physicians’ work–life balance worldwide, the literature is limited in regards to physicians in the Middle East.

Over the past two decades, research on physicians’ job satisfaction (JS) has grown in importance [8,11]. The outcomes of JS extend beyond physicians’ well-being to their mental health, the quality of care given to patients, and their satisfaction [12]. In addition, the conflict between work and family also has a negative impact on physicians’ JS, which may reflect on the doctor–patient relationship [8]. In light of the detrimental impact work–life conflict has on individuals’ lives, combined with the increased risk of physicians suffering from it and the limited data on the levels of WLB and JS among physicians in Jordan and the Middle East, the aim of the study was to assess the status of WLB, JS, and life satisfaction (LS) among physicians practicing in Jordan. This was achieved by analyzing the three indices in addition to how various demographic, professional, and academic correlates impacted each one.

## 2. Methods

### 2.1. Study Design and Population

This study utilized a cross-sectional observational design that took place between August 2021 and April 2022. This was achieved by sharing a Google Forms questionnaire with any physician practicing in Jordan who had completed their internship year. The questionnaire was distributed broadly among physicians nationwide, in any private, public, university, or military healthcare setting. The study included 625 practicing physicians across Jordan.

### 2.2. Study Tool

The survey consisted of 37 detailed self-report questions covering seven main categories. The first was demographic information: sex, age, marital status, area of residence, and number of children (five questions). The second asked about professional and academic information: working sector, position, number of years practicing, department, monthly income, hours per week, and calls per month (seven questions).

The third, fourth, and fifth categories were taken from a validated 15-item WLB scale survey constructed by Hayman utilizing a seven-point time related scale (e.g., 1, not at all; 4, sometimes; and 7, all the time) [13]. The third category consisted of seven questions addressing the impact of work on personal life (WIPL). The fourth consisted of four questions addressing the impact of personal life on work (PLIW). The fifth area consisted of four questions addressing the work/personal life enhancement (WPLE). The sixth category, in which the Andrew and Withney Job Satisfaction scale was utilized, used a seven-point Likert scale (from 1 (delighted) to 7 (not at all satisfied)) [14]. The seventh was the Satisfaction with Life Scale (SWLS) developed by Diener et al., which used a seven-point Likert scale (from 1 (strongly disagree) to 7 (strongly agree)) [15]. A higher work–life score was interpreted as a negative WLB, while higher JS and LS scores were interpreted as positive JS and LS. Prior to the distribution of the survey, a pilot study of 20 individuals was performed. All subscales were found to have a Cronbach’s alpha >0.7.

### 2.3. Data Collection Procedure

Physicians across Jordan were asked to complete an anonymous, free online survey over a period of 8 months. The sample size was determined using a structural equation model (SEM) through an open-source epidemiological calculator (OpenEpi: Open Source Epidemiologic Statistics for Public Health, Version. www.OpenEpi.com, accessed on 12 June 2021), in addition to previous papers tackling similar topics in Jordan. To increase the sample size, the questionnaire was distributed through a number of social media platforms, and participants were asked to share it with their friends and social networks. The questionnaire took 10 min to complete in the English language. The English language is the official language of communication in the medical field in Jordan.

Ethical approval was granted by the Institutional Review Board (IRB) of Jordan University Hospital, and the study was conducted in accordance with the Declaration of Helsinki. Participation in this study was elective, without any form of compensation, and the study was conducted after formal consent was obtained from the participants.

### 2.4. Statistical Analysis

SPSS version 25.0 (IBM, Chicago, IL, USA) was used to perform the data analyses. First, we described the characteristics of the sample using counts and percentages for categorical variables and medians and interquartile ranges (IQRs) for continuous variables. Next, we tested the relationships between a subset of characteristics and the WLB score, JS score, and LS score (measured on a continuous scale) using the Mann–Whitney U test (for categorical variables with two groups), Kruskal–Wallis H test (for categorical variables with three or more groups), and Spearman’s ρ (for continuous variables). If the Kruskal–Wallis H test was statistically significant, we performed pairwise comparisons using the Mann–Whitney U test and corrected for multiple testing using the Benjamini–Hochberg method. We interpreted values of *p* ≤ 0.05 to indicate statistical significance.

## 3. Results

### 3.1. Characteristics of the Sample

We included 625 physicians in the final analysis. The characteristics of all physicians are summarized in Table 1. The majority of our sample (59.0%) were male and ranged in age from 24 to 75, with a median of 31 (12.5). Most of the participants were married (61.6%), yet nearly half (49.0%) had no children; the median number of children was one (three). Among the participants, 46.7% reported having a chronic disease, most commonly hypertension. Amman, the capital of Jordan and its most populous city, was the area of residence of the overwhelming majority (74.7%), followed by Irbid (8.0%), a major city located in the northernmost part of Jordan, then Balqa (4.8%), a mid-sized city in the center of Jordan near Amman.

Concerning specialty, our sample included 19 specialties, with general surgery, obstetrics and gynecology, and internal medicine being the three highest specialties, in that order. Nearly a third (32.3%) of the participants worked in the private sector, followed by the Ministry of Health (27.5%) and university hospitals (23.5%). Concerning workload, the median number of weekly working hours was 48 (32), while the median number of calls per month was six (seven). Among our participants, the mean number of years practicing medicine was six (11.5). Most of the participants (47.7%) had a monthly income of JOD 500-1000 (USD 700-1400), which coincides with 41.3% of our sample being at a resident level.

### 3.2. WLB among Physicians

Table 2 illustrates the distribution of the responses to each item on the WLB scale. Overall, 62.9% of the participants were found to have a work–life conflict. Among participants, 39% had frequent work interference with personal life, with missing personal activities because of work being the most common conflict (66.8%), followed by personal life suffering from work (63.2%) and work causing personal life to be difficult (61%). As for personal life interference with work, only 6.2% felt that their work suffered from their personal life, and the most common conflict was being too tired to be effective at work. Interestingly, only 9.4% responded that they frequently felt their work was affected by personal life, and 8.8% felt that personal matters made it hard to work. As for work–personal life enhancement, 37% of participants reported having negative work–personal life enhancement. Of note, three-fourths of respondents (75.0%) reported that the COVID-19 pandemic affected their WLB.

### 3.3. Factors Affecting WLB

We investigated the relationships between a subset of characteristics and the WLB score (Table 3). The median score was higher in females (*p* = 0.001) and those who were single (*p* = 0.003). The median score was lower for physicians who worked in the private sector (*p* < 0.001), consultants (*p* < 0.001), and those that made more than JOD 2,000 per month (*p* < 0.001). In addition, differences in WLB existed between specialties (*p* = 0.001). The WLB score was negatively correlated with age (ρ = −0.3; *p* < 0.001), number of children (ρ = −0.3; *p* < 0.001), and years practicing medicine (ρ = −0.3; *p* < 0.001). On the other hand, it was positively correlated with number of hours per week (ρ = 0.4; *p* < 0.001) and number of calls (ρ = 0.2; *p* < 0.001).

### 3.4. JS and LS among Physicians

Table 4 and Table 5 show the distributions of the responses on the JS and LS scales, respectively. Regarding JS, 19% of participants had a score indicating positive JS, while 22.1% had a score indicating a negative JS. The most positive responses were for the “How do you feel about the work you do on your job (the work itself)?” and “How do you feel about the people you work with (your co-workers)?” JS questions, with 51% and 41%, respectively. Meanwhile, “How do you feel about what you have available during your job (equipment, information, good supervision, and so on)?” and “What is it like where you work (the physical surroundings, the hours, the amount of work you are asked to do)?” had the most negative responses, with 44.7% and 41%, respectively.

Regarding LS, 20.5% disagreed with the statements indicating LS, while 37% agreed. Nearly half of the respondents (53.1%) disagreed with the statement “In most ways, my life is close to my ideal”, and 58.1% disagreed with the statement “If I could live my life over, I would change almost nothing.” Conversely, the statements “I am satisfied with my life” and “So far, I have gotten the important things I want in life” were the most agreed upon, with 41% of respondents each.

### 3.5. Factors Affecting JS and LS

We also investigated the relationships between a subset of characteristics and JS and LS scores (Table 6). Regarding JS, the median score was higher in males (*p* = 0.02), those who were married (*p* < 0.001), those who were working in the private sector (*p* < 0.001), consultants (*p* < 0.001), and those who had a monthly income higher than JOD 2000 (*p* < 0.001). The JS score was positively correlated with age (ρ = 0.363; *p* < 0.001), number of children (ρ = 0.344; *p* < 0.001), and years practicing medicine (ρ = 0.368; *p* < 0.001). A negative correlation was found with number of hours per week (ρ = −0.315; *p* < 0.001) and number of on-calls (ρ = −0.215; *p* < 0.001).

Similarly, in LS, the median score was higher in those who were married, those who worked in the private sector, consultants, and those with a monthly income higher than JOD 2000 (*p* < 0.001). As for correlations, the LS score was also positively correlated with age (ρ = 0.338; *p* < 0.001), number of children (ρ = 0.342; *p* < 0.001), and years practicing medicine (ρ = 0.368; *p* < 0.001). The LS score was negatively correlated with number of hours per week (ρ = −0.333; *p* < 0.001) and number of on-calls (ρ = −0.201; *p* < 0.001).

### 3.6. Relationship between WLB, JS, and LS

A correlation was found between the three scores. The WLB score was negatively associated with both JS (ρ = −0.583; *p* < 0.001) and LS (ρ = −0.609; *p* < 0.001), while JS and LS were strongly positively correlated (ρ = 0.678; *p* < 0.001).

## 4. Discussion

The main finding of our study was that around two-thirds of participants had a work–life conflict. In addition, approximately four out of ten physicians had frequent work interference with personal life. The WLB was lower in females, those who were single, those with a higher number of working hours, and those with a higher number of on-call days. On the other hand, the WLB was higher for those who were older, who had a higher number of children, increased years of practicing medicine, private sector workers, consultants, and those with a monthly income of more than JOD 2000. Regarding specialties, obstetrics and gynecology, followed by family medicine, general surgery, and internal medicine, showed a lower WLB. Regarding JS and LS, they were higher among those who were older, married, consultants, working in the private sector, had more children, had an increased number of years practicing medicine, and had a monthly income of more than JOD 2000. JS and LS were lower in participants with increased working hours and an increased number of calls. JS was higher among males, but there was no difference in overall LS between the genders. JS and LS increased with an increase in the WLB.

In a systematic review in the Middle East, Chemali et al. reported that the burnout prevalence range was between 40 and 60% among healthcare workers [16]. Jordan exhibited even greater prevalence values than those reported in the previous literature. According to a cross-sectional study of 481 resident physicians in Jordan, 77% of them were found to have burnout [17]. Similarly, Al-Taher et al. found that 53.6% of Jordanian resident physicians had a high grade of emotional exhaustion, with 82.4% exceeding the 24 h shift length [18]. Moreover, it was found that physicians had a greater likelihood of exhibiting higher burnout scores and less WLB than the general population in the United States [4,5]. Given that there have been no previous studies on WLB, JS, or LS in our area, it is difficult to compare our findings with the literature.

The negative consequences of work–life conflict and low quality of life among healthcare employees, particularly resident physicians, are well documented and include an increase in the likelihood of medical errors, burnout, lower care quality, and patient dissatisfaction [19,20,21,22]. In a review by Amoafo et al., they found that younger-aged physicians, females, and negative marital status were predictors of burnout [7]. Our study showed similar findings since younger-aged, single, or female physicians had a lower WLB. In addition, resident physicians had the least WLB, JS, and LS compared with other positions. This is especially alarming since previous research has demonstrated that a lack of WLB during postgraduate medical training has a negative effect on physicians’ learning, progression, and well-being [23]. As the ramifications of the lifestyle of healthcare practitioners persist, married doctors experience an increased work–family conflict, especially in internal medicine, surgery, obstetrics and gynecology, and pediatrics departments [8]. As a result, it is more difficult for young physicians to get married, have children, and maintain a work–family balance [8].

A recent review showed there has been a shift in obstetrics and gynecology in recent years, as a growing number of women express a desire for a better WLB [24]. In addition, it has been reported previously that the obstetrics and gynecology field has one of the lower rates of burnout in the medical profession [5]. However, our study found that the lowest WLB and JS were reported by obstetrics and gynecology, followed by family medicine. In addition, a previous study on Jordanian resident physicians found that being an obstetrics and gynecology resident had the highest burnout levels and was a significant predictor of higher levels of burnout [17]. This could be due to the irregular working hours, paperwork, delays in referrals to specialists and test results, lack of respect and appreciation, lack of support, complex patients, and lack of WLB [5,25].

As expected, increasing WLB was positively correlated with JS and LS, as reported by previous studies [26]. In general, JS was higher than LS, which could be explained by the fact that despite working their desired job, nearly a half of the participants had a monthly income of JOD 500–1000 (USD 700–1400) and worked an increased number of hours. This, in turn, negatively affected their lifestyles and time spent with their families. A previous study showed that lower wages were one of the most important factors affecting JS [27], which is comparable to our results, as JS and LS were higher in those with a monthly income of more than JOD 2000. Our study found that males had higher JS, and there was no difference in LS, which contradicts the findings of several prior studies that found no differences between females and males in terms of JS [28,29,30].

The main strength of our study is that it is the first in-depth article to tackle WLB among physicians in Jordan. In addition, the survey assessed various validated scales, including the WLB score, the JS score, and the LS score. We included physicians at multiple levels, as well as workers from different working sectors, allowing us to estimate the desired scores and generalize our findings. Moreover, using a validated and self-administered questionnaire reduced the interviewer bias. Therefore, we believe that our findings could accurately reflect the overall WLB, JS, and LS in Jordan. The authors acknowledge that this study is not without limitations. The study’s cross-sectional design limits our ability to determine causal effects. Nevertheless, most of our results are consistent with causal relations asserted by previously published research. In addition, a non-random convenience sampling method was used to recruit participants due to the low participation rate. Moreover, because our study relied on self-reported data, response bias was a limitation. Our research was focused on the five main disciplines (general surgery, obstetrics and gynecology, internal medicine, orthopedics, and family medicine); thus, our conclusions may not apply to other specialties or subspecialties. Further additional regional studies to elucidate these concerns in greater depth are required.

Our study demonstrates that work–life conflict is highly prevalent among Jordanian physicians and highlights the significance of WLB in supporting physicians’ well-being and performance. Priority should be given to expanding national initiatives to investigate and reduce work-related stress by enhancing medical professionals’ mental health and working environments. This is especially important since the negative impact of poor WLB and increased stress extends beyond the doctor himself to putting the patients at risk of unintentional harm, increasing the number of errors made by the doctors, and affecting the ongoing education process of the resident physicians. Further studies studying the implementation of initiatives aimed at improving WLB should be conducted.

## Figures and Tables

**Table 1:** Characteristics of (*n* = 625) physicians.

Characteristic	*n* (%) or Median (IQR)
**Age (years)**	31 (12.5)
**Sex**	
Male	369 (59.0)
Female	256 (41.0)
**Marital status**	
Married	385 (61.6)
Single	235 (37.6)
Divorced	5 (0.8)
**Number of children**	1 (3.0)
**Area of residence**	
Amman	468 (74.6)
Irbid	51 (8.1)
Balqa	40 (4.8)
Zarqa	24 (3.8)
Other	42 (6.72)
**Chronic disease**	
Yes	292 (46.7)
No	333 (53.3)
**Working sector**	
Private sector	202 (32.2)
Ministry of Health	172 (27.4)
University hospital	149 (23.8)
Specialized medical center	104 (16.6)
**Position**	
General practitioner	98 (15.6)
Resident	258 (41.1)
Fellow	19 (3.0)
Specialist	118 (18.8)
Consultant	134 (21.4)
**Specialty**	
General surgery	87 (13.9)
Obstetrics and gynecology	79 (12.6)
Internal medicine	77 (12.3)
Orthopedics	56 (9.0)
Family medicine	50 (8.0)
Other	276 (44.2)
**Monthly income**	
Unpaid	49 (7.8)
Less than JOD 500	48 (7.7)
JOD 500–1000	298 (47.7)
JOD 1000–2000	101 (16.2)
More than JOD 2000	129 (20.6)
**Years practicing medicine**	6 (11.5)
**Number of hours working per week**	48 (32.0)
**Number of on call days per month**	6 (7.0)

1 JOD = 1.40 USD.

**Table 2:** Distribution of responses (*n* = 625) on the work–life balance scale.

Statements	*n* (%)						
	Not at All	Very Rarely	Rarely	Sometimes	Frequently	Very Frequently	All the Time
**WIPL**							
My personal life suffers because of work.	24 (3.8)	13 (2.1)	29 (4.6)	164 (26.2)	166 (26.6)	120 (19.2)	109 (17.4)
My job makes personal life difficult.	29 (4.6)	11 (1.8)	42 (6.7)	162 (25.9)	156 (25.0)	121 (19.4)	104 (16.6)
I neglect personal needs because of work.	29 (4.6)	23 (3.7)	51 (8.2)	165 (26.4)	168 (26.9)	122 (19.5)	67 (10.7)
I put personal life on hold for work.	29 (4.6)	23 (3.7)	47 (7.5)	154 (24.6)	175 (28.0)	132 (21.1)	65 (10.4)
I miss personal activities because of work.	24 (3.8)	18 (2.9)	30 (4.8)	136 (21.8)	171 (27.4)	145 (23.2)	101 (16.2)
I struggle to juggle work and non-work activities.	27 (4.3)	17 (2.7)	46 (7.4)	181 (29.0)	189 (30.2)	91 (14.6)	74 (11.8)
I am happy with the amount of time for non-work activities.	47 (7.5)	35 (5.6)	82 (13.1)	136 (21.8)	118 (18.9)	67 (10.7)	140 (22.4)
**PLIW**							
My personal life drains me of energy for work.	93 (14.9)	54 (8.6)	123 (19.7)	195 (31.2)	97 (15.5)	38 (6.1)	25 (4.0)
I am too tired to be effective at work.	67 (10.7)	53 (8.5)	122 (19.5)	194 (31.0)	98 (15.7)	58 (9.3)	33 (5.3)
My work suffers because of my personal life.	175 (28.0)	127 (20.3)	163 (26.1)	101 (16.2)	37 (5.9)	12 (1.9)	10 (1.6)
It is hard to work because of personal matters.	173 (27.7)	112 (17.9)	151 (24.2)	134 (21.4)	27 (4.3)	23 (3.7)	5 (0.8)
**WPLE**							
My personal life gives me energy for my job.	49 (7.8)	48 (7.7)	92 (14.7)	207 (33.1)	128 (20.5)	56 (9.0)	45 (7.2)
My job gives me energy to pursue personal activities.	146 (23.4)	84 (13.4)	165 (26.4)	139 (22.2)	55 (8.8)	19 (3.0)	17 (2.7)
I have a better mood at work because of personal life.	94 (15.0)	45 (7.2)	98 (15.7)	192 (30.7)	120 (19.2)	41 (6.6)	35 (5.6)
I have a better mood because of my job.	138 (22.1)	65 (10.4)	132 (21.1)	144 (23.0)	88 (14.1)	41 (6.6)	35 (5.6)

WIPL = work impact on personal life, PLIW = personal life impact on work, WPLE = work personal life enhancement.

**Table 3:** Work–life balance correlates of (*n* = 625) physicians.

Characteristic	Median WLB Score (IQR) or Spearman’s ρ	*p* Value
**Age (years)**	−0.310	<0.001
**Sex**		0.001
Male	4.2 (1.1)	
Female	4.4 (1.0)	
**Marital status**		0.003
Married	4.2 (1.1)	
Single	4.4 (1.1)	
Divorced	4.5 (1.8)	
**Number of children**	−0.269	<0.001
**Chronic disease**		0.105
Yes	4.3	
No	4.2	
**Working sector**		<0.001
Private sector	4.1(1.2)	
Ministry of Health	4.5 (1.0)	
University hospital	4.3 (1.1)	
Specialized medical center	4.3 (0.8)	
**Position**		<0.001
General practitioner	4.3 (0.8)	
Resident	4.5 (1.0)	
Fellow	4.3 (0.9)	
Specialist	4.3 (0.8)	
Consultant	3.7 (1.2)	
**Specialty**		<0.001
General surgery	4.3 (1.1)	
Obstetrics and gynecology	4.6 (0.9)	
Internal medicine	4.3 (1.0)	
Orthopedics	4.0 (1.1)	
Family medicine	4.4 (1.1)	
Other	3.9 (1.4)	
**Monthly income**		<0.001
Unpaid	4.3 (0.8)	
Less than JOD 500	4.2 (1.0)	
JOD 500–1000	4.5 (1.0)	
JOD 1000–2000	4.2 (1.0)	
More than JOD 2000	3.7 (1.2)	
**Years practicing medicine**	−0.318	<0.001
**Number of hours working per week**	0.401	<0.001
**Number of on call days per month**	0.231	<0.001

1 JOD = 1.40 USD.

**Table 4:** Distribution of responses (*n* = 625) on the job satisfaction scale.

Statements	*n* (%)						
	Terrible	Unhappy	Mostly Dissatisfied	Mixed	Mostly Satisfied	Pleased	Delighted
How do you feel about your job?	42 (6.7)	63 (10.1)	78 (12.5)	237 (37.9)	122 (19.5)	59 (9.4)	24 (3.8)
How do you feel about the people you work with (your co-workers)?	26 (4.2)	51 (8.2)	70 (11.2)	222 (35.5)	157 (25.1)	79 (12.6)	20 (3.2)
How do you feel about the work you do on your job (the work itself)?	32 (5.1)	50 (8.0)	51 (8.2)	173 (27.7)	176 (28.2)	107 (17.1)	36 (5.8)
What is it like where you work (the physical surroundings, the hours, the amount of work you are asked to do)?	78 (12.5)	70 (11.2)	108 (17.3)	221 (35.4)	97 (15.5)	43 (6.9)	8 (1.3)
How do you feel about what you have available during your job (equipment, information, good supervision, and so on)?	69 (11.0)	114 (18.2)	97 (15.5)	185 (29.6)	107 (17.1)	46 (7.4)	7 (1.1)

**Table 5:** Distribution of responses (*n* = 625) on the life satisfaction scale.

Statements	*n* (%)						
	Strongly Disagree	Disagree	Slightly Disagree	Neutral	Slightly Agree	Agree	Strongly Agree
In most ways, my life is close to my ideal.	105 (16.8)	151 (24.2)	76 (12.2)	154 (24.6)	66 (10.6)	58 (9.3)	15 (2.4)
The conditions of my life are excellent.	72 (11.5)	132 (21.1)	79 (12.6)	155 (24.8)	82 (13.1)	88 (14.1)	17 (2.7)
I am satisfied with my life.	53 (8.5)	98 (15.7)	70 (11.2)	148 (23.7)	89 (14.2)	131 (21.0)	36 (5.8)
So far, I have gotten the important things I want in life.	54 (8.6)	116 (18.6)	64 (10.2)	135 (21.6)	100 (16.0)	124 (19.8)	32 (5.1)
If I could live my life over, I would change almost nothing.	109 (17.4)	167 (17.4)	87 (13.9)	85 (13.6)	67 (10.7)	81 (13.0)	29 (4.6)

**Table 6:** Job satisfaction and life satisfaction correlates of (*n* = 625) physicians.

Characteristic	Median JS Score (IQR) or Spearman’s ρ	*p* Value	Median LS Score (IQR) or Spearman’s ρ	*p* Value
**Age (years)**	0.363	<0.001	0.338	<0.001
**Sex**		0.02		0.221
Male	4.2 (1.6)		3.8 (2.2)	
Female	3.8 (1.2)		3.4 (2.2)	
**Marital status**		<0.001		<0.001
Married	4.2 (1.4)		4.0 (2.4)	
Single	3.8 (1.4)		3.2 (1.8)	
Divorced	3.8 (2.1)		3.2 (2.9)	
**Number of children**	0.344	<0.001	0.342	<0.001
**Chronic disease**		0.327		0.082
Yes	3.8 (1.4)		3.4 (2.6)	
No	4.0 (1.6)		3.8 (2.0)	
**Working sector**		<0.001		<0.001
Private sector	4.4 (1.4)		4.0 (2.8)	
Ministry of Health	3.6 (1.2)		3.4 (2.0)	
University hospital	3.8 (1.4)		3.8 (2.2)	
Specialized medical center	4 (1.5)		3.3 (2.0)	
**Position**		<0.001		<0.001
General practitioner	3.8 (1.2)		3.4 (2.1)	
Resident	3.6 (1.3)		3.2 (1.8)	
Fellow	4.0 (0.8)		3.8 (1.6)	
Specialist	4.0 (1.4)		3.2 (2.3)	
Consultant	4.9 (1.4)		5.0 (1.9)	
**Specialty**		<0.001		0.083
General surgery	4.2 (1.4)		3.6 (2.8)	
Obstetrics and gynecology	3.6 (1.4)		3.2 (2.0)	
Internal medicine	4.0 (1.7)		3.6 (2.0)	
Orthopedics	4.4 (1.6)		3.8 (2.0)	
Family medicine	3.8 (1.7)		3.8 (2.4)	
Other	3.8 (1.9)		3.6 (2.1)	
**Monthly income**		<0.001		<0.001
Unpaid	3.8 (1.2)		3.6 (2.0)	
Less than JOD 500	3.7 (1.2)		2.8 (1.9)	
JOD 500–1000	3.6 (1.4)		3.2 (1.8)	
JOD 1000–2000	4.2 (1.2)		3.8 (2.2)	
More than JOD 2000	4.8 (1.2)		5.0 (1.9)	
**Years practicing medicine**	0.368	<0.001	0.368	<0.001
**Number of hours working per week**	−0.315	<0.001	−0.333	<0.001
**Number of on call days per month**	−0.215	<0.001	−0.201	<0.001

1 JOD = 1.40 USD.

## Data Availability

The data presented in this study are available upon request from the corresponding author. The data are not publicly available due to privacy concerns.
